# Prevalence of *Rickettsia spp*. in Ticks and Serological and Clinical Outcomes in Tick-Bitten Individuals in Sweden and on the Åland Islands

**DOI:** 10.1371/journal.pone.0166653

**Published:** 2016-11-15

**Authors:** Anders Lindblom, Katarina Wallménius, Johanna Sjöwall, Linda Fryland, Peter Wilhelmsson, Per-Eric Lindgren, Pia Forsberg, Kenneth Nilsson

**Affiliations:** 1 Unit of Infectious Diseases, Department of Medical Sciences, Uppsala University, Uppsala, Sweden; 2 Unit of Clinical Bacteriology, Department of Medical Sciences, Uppsala University, Uppsala, Sweden; 3 Department of Infectious Diseases, Linköping University, Linköping, Sweden; 4 Clinical Immunology, Department of Clinical and Experimental Medicine, Linköping University, Linköping, Sweden; 5 Medical Microbiology, Department of Clinical and Experimental Medicine, Linköping University, Linköping, Sweden; 6 Medical Services, County Hospital Ryhov, Jönköpng, Sweden; 7 Department of Infectious Diseases and Department of Clinical and Experimental Medicine, Linköping University, Linköping, Sweden; 8 Center of Clinical Research, Dalarna, Falun, Sweden; Onderstepoort Veterinary Institute, SOUTH AFRICA

## Abstract

Tick-transmitted diseases are an emerging health problem, and the hard tick *Ixodes ricinus* is the main vector for *Borrelia* spp., tick-borne encephalitis virus and most of the spotted fever *Rickettsiae* in Europe. The aim of the present study was to examine the incidence of rickettsial infection in the southernmost and south central parts of Sweden and the Åland Islands in Finland, the risk of infection in humans and its correlation with a bite of a *Rickettsia*-infected tick, the self-reported symptoms of rickettsial disease, and the prevalence of co-infection between *Rickettsia* spp. and *Borrelia* spp. Persons with a recent tick bite were enrolled through public media and asked to answer a questionnaire, provide a blood sample and bring detached ticks at enlistment and at follow-up three months later. Blood samples were previously analysed for *Borrelia* spp. antibodies and, for this report, analysed for antibodies to *Rickettsia* spp. by immunofluorescence and in 16 cases also using Western Blot. Ninety-six (44.0%) of the 218 participants were seropositive for IgG antibodies to *Rickettsia* spp. Forty (18.3%) of the seropositive participants had increased titres at the follow-up, indicating recent/current infection, while four (1.8%) had titres indicating probable recent/current infection (≥1:256). Of 472 ticks, 39 (8.3%) were *Rickettsia* sp. positive. Five (31.3%) of 16 participants bitten by a *Rickettsia*-infected tick seroconverted. Experience of the self-reported symptoms nausea (p = 0.006) and radiating pain (p = 0.041) was more common among those with recent, current or probable infection compared to those who did not seroconvert. Participants who showed seroreactivity or seroconversion to *Rickettsia* spp. had more symptoms than those who were seronegative. Seven (3.2%) participants showed seroconversion to *Borrelia* spp., and three (1.4%) of these showed seroconversion to both *Rickettsia* spp. and *Borrelia* spp., in accordance with previous studies in Sweden. Symptoms of rickettsial disease were in most of the cases vague and general that were difficult to differentiate from other tick-borne diseases.

## Introduction

Several tick-transmitted microorganisms can cause human disease, among them members of the *Borrelia burgdorferi* sensu lato group, including *B*. *afzelii*, *B*. *garinii*, tick-borne encephalitis virus, *Anaplasma* spp., *Candidatus* Neoehrlichia mikurensis, and most of the species of the spotted fever group of rickettsiae (SFGR). The main vector in Sweden and other European countries is the hard tick, *Ixodes ricinus*. At least 22 species of SFGR are known, 17 of which are identified as human pathogens [1]. The predominant SFG *Rickettsia* in ticks in Sweden is *Rickettsia helvetica*, which appears at a prevalence between 1.7–22% in ticks collected from different areas, whereas *R*. *sibirica* has in one occasion also been detected in *I*. *ricinus* ticks in Sweden [2, 3]. *R*. *helvetica* infection is primarily considered a self-limited disease with fever, myalgia and headache, in some cases with a rash and eschar. However, the infection can present more severe symptoms, including neurological symptoms as well as perimyocarditis [4–12]. Only a limited number of patients with serology-based diagnosis have been reported to date. For this reason, more studies are needed to understand all clinical manifestations of *R*. *helvetica* [1]. In addition to *R*. *helvetica*, *R*. *felis*, whose main vector is the cat flea (*Ctenophalides felis*), has also been associated with meningitis [13].

Ticks may harbour and be simultaneously infested with many microorganisms [14–16]. In a study from Italy, the most frequently observed double infestation in ticks was SFG *Rickettsiae* and *Borrelia burgdorferi* sensu lato [17]. Despite the fact that several agents appear in ticks, co-infections in humans have rarely been studied, although a few surveys have been conducted that included co-infections with different *Rickettsia* species [18–23].

The aims of the present study were: 1) to examine the prevalence of rickettsial disease in humans with a confirmed tick bite, 2) to calculate the risk of infection after being bitten by a *Rickettsia* infected tick, 3) to describe the clinical manifestations of rickettsial disease, and 4) to chart the extent to which a serological response to both *Rickettsia* spp. and *Borrelia* spp. occurs following a tick bite.

## Materials and Methods

### Patients

The Tick-Borne Disease (TBD) STING study is a prospective study following recently tick-bitten individuals for a 3-month study period [24,25]. Individuals ≥ 18 years of age with an observed and recent tick bite were asked through local public media to bring the tick, after detachment, to their Primary Health Care (PHC) centre. In the present study, a total of 222 participants were enrolled from May 2008 to September 2009. Four participants were excluded from the study: two were being treated with antibiotics at the time of enrolment and two failed to show up for the follow-up visit. All participants who visited the PHC centre were, after giving their written informed consent, asked to complete a questionnaire, to provide a blood sample and to donate their ticks for further investigation. They were followed up with a second visit to the PHC centre three months later, at which time they completed a second questionnaire, provided a new blood sample and brought ticks from additional tick bites. All samples were transported to Linköping University and frozen within 3 days at -70°C for later analyses.

**Fig 1:**
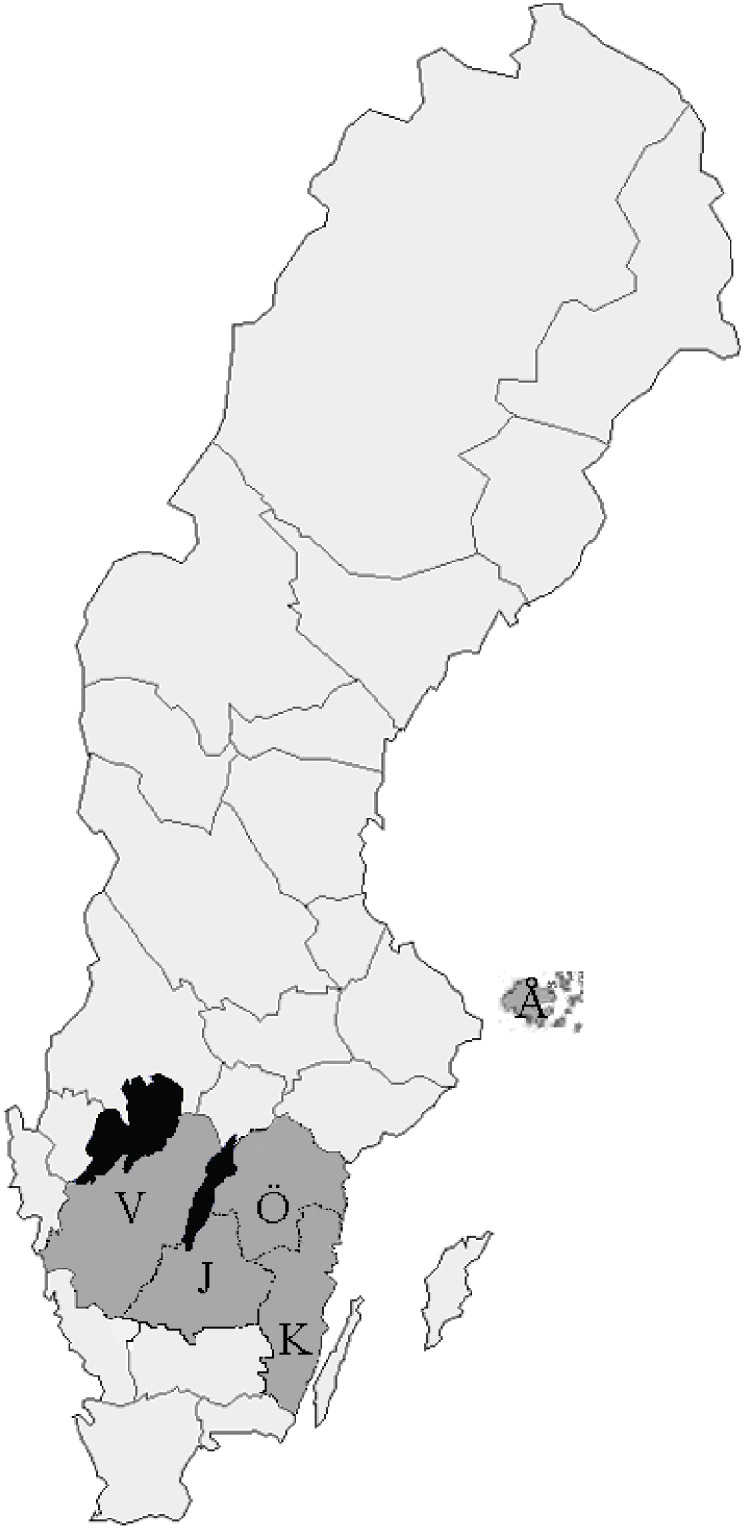
Map showing the areas where participants were recruited. V = Västra Götaland; Ö = Östergötland; J = Jönköping County; K = Kalmar County; Å = Åland Islands.

The questionnaire completed at enrolment included information about the number of tick bites, geographical location at which the tick bite occurred and medical history regarding previous tick-borne diseases [26]. The second questionnaire completed at the follow-up visit included questions about new tick bites, non-specific symptoms associated with tick-borne diseases and whether the participants had sought medical care. The symptoms listed included fatigue, headache, loss of appetite, weight loss, nausea, fever, neck pain, vertigo myalgia/arthralgia, numbness, radiating pain and cognitive difficulties. Medical records were examined to determine whether the patients had been diagnosed with a tick-borne infection. The regions participating in the current study were located in the south central parts of Sweden represented by: Västra Götaland (Lidköping); Östergötland (Söderköping; Kisa; Vikbolandet) Jönköping County (Bankeryd), southernmost Sweden (Kalmar) and the Åland Islands in Finland (Fig 1). These areas were selected because they are known to be high endemic tick areas identified in previous studies [24, 25].

### Ticks

A total of 472 ticks detached from the participants were collected from the different locations (98 ticks in Lidköping, 97 in Jönköping, 98 in Östergötland, 82 in Kalmar and 97 on the Åland Islands). Some participants delivered additional detached ticks during the 3-month follow-up period. From participants who seroconverted and presented a *Rickettsia* negative tick at inclusion, additional ticks were analysed using PCR to determine whether infection occurred after recruitment. Ticks were photographed and measured dorsally and ventrally to determine species; all belonged to *I*. *ricinus* [24, 25, 27]. Life cycle stages of the ticks were determined. Blood feeding time of nymphs and female adults were estimated using the scutal and coxal indices [28].

### Serology (Immunofluorescence assay)

*R*. *helvetica*-infected Vero cells supplemented with 10% yolk sac solution were used as antigen. The antigen solution was applied to each well of the microscope slides, dried, and fixed in acetone, where after the serum was serially diluted in titres 1/64, 1/128 and 1/256, respectively, and incubated as previously described [18]. Human blood donor serum was used as a negative control, and serum from a patient with proven end-point titres of 1:256 and 1:128 of IgG/IgM, respectively, was used as a positive control. Fluorescein isothio-cyanate-conjugated (FITC) γ–and mu-chain-specific polyclonal rabbit anti-human IgG and IgM (Ref.: F0202 and FF0203, Dako, Denmark) were used for detection of IgG and IgM antibodies, the latter after pretreatment with rheumatoid factor absorbent (Immunkemi, Stockholm, Sweden). The participants were divided into four groups based on their serologic results. As done at the *Rickettsia* laboratory in Marseille (Unité des Rickettsies), IgG titres ≥ 128 was judged as indicative of infection and a titre ≥ 1:256 was assumed to be the result of a current rather than a past infection [29]. Group 1 (confirmed case): four-fold or greater rise in IgG titre between the first and second sera. Group 2 (probable case): single or repeatable IgG endpoint titres of ≥1:256 were regarded as evidence of a recent or current infection. Group 3 (seroreactive): IgG and/or IgM titres ≥ 1:64 and ≤ 1:128 were considered indicative of either past infection or early response (IgM) to infection, while persisting IgG titres with or without IgM reactivity were considered to indicate a past infection. Persisting IgM antibodies alone were interpreted as non-specific cross-reactivity due to exposure to other organisms or autoimmune responses or possibly as a sign of previous exposure [30]. Group 4: IgG/IgM titres <1:64 were considered negative. All groups were evaluated separately. However, for the evaluation of symptoms, we combined the serological groups with findings on rickettsial DNA in the ticks.

In previous studies, sera from the participants were examined regarding *B*. *burgdorferi*, as described here [24, 25].

### Western Blot (WB)

Sera from 16 of the IgG-positive participants (Pat. nos. K7, K9, K46, K56, L16, L43, L46, S71. S72, S75, V6, Å16, Å23 and Å35 (Group 1) and K13 and Å13 (Group 2) were diluted to titres 1:200 and tested for WB with *R*. *helvetica* whole-cell antigen using Amersham WB system (GE Healthcare) with the secondary antibody Antihuman IgG DyLight^™^549 (Rockland Inc. cat.no 609-142-123) in the concentration of 1:10,000 in accordance with the manufacturer’s instructions. A serum from a patient with a proven end-point IgG/IgM of 1:512/1:128 to *R*. *helvetica* was used.with served as the positive control. The secondary antibody alone served as the negative control together with serum sample from a healthy blood donor

### PCR

All cDNA samples from the ticks were individually assayed using a real-time PCR targeting the citrate synthase (*gltA*) gene of *Rickettsia* spp., as previously described [2, 14, 27]. Two to five μl cDNA was used as a template in each reaction, together with 0.25 μl LC Uracil-DNA glycosylase (UNG) (Roche Diagnostics, Mannheim, Germany) to minimize the risk of contamination. The reactions were run in a Rotor-Gene 3000 (Qiagen, Sydney, Australia) using LightCycler^®^ TaqMan^®^ Master (Roche Diagnostics, Mannheim, Germany). In each amplification trial, a negative control, sterile water and a positive standard plasmid constructed by cloning the 74 bp PCR product of the *glt*A gene into a PCR 4-TOPO vector (TOPO^®^ TA Cloning^®^ kit for Sequencing, Invitrogen, Carlsbad, CA, USA) and containing the cloned 74 bp fragment of the *gltA* gene were included in 10-fold serial dilutions. All samples that were positive in real-time PCR were further amplified for analysis of a fragment of the genes coding for the outer membrane protein B, *ompB*, 17-kDa or using a semi-nested PCR targeting the *gltA* gene, as previously described [2, 14]. All PCR products considered for sequencing were cleansed using Exonuclease I and FastAP^™^ Thermosensitive Alkaline Phosphatase (Fermentas GmbH). Sequencing analysis of PCR products was performed at Macrogen Inc. (Macrogen Europe, Amsterdam, Netherlands). DNA Baser version 2.80.0 (HeracleSoftware, Lilienthal, Germany) and BioEdit Sequence Alignment Editor Version 7.0.5.3 (Ibis Therapeutics, Carlsbad, CA) were used for sequence alignments. For species identification, sequences were examined using the Basic Local Alignment Search Tool (BLAST). A summary of primers, probe, sequences and products is provided in Table 1.

**Table 1:** Details of primers, probe, nucleotide sequences, gene positions product sizes in PCR assays and sequencing of rickettsial genes [2].

Gene	Primers and probe	Nucleotide sequence (5´to 3`)	Product size (bp)
*gltA*	CS-F	TCG CAA ATG TTC ACG GTA CT	74
	CS-R	TCG TGC ATT TCT TTC CAT TGT G	
	CD-P	6-FAM-TGC AAT AGC AAG AAC CGTAGG CTG GAT G—BBQ-1	Probe
*ompB*	Rc.rompB.4362p	GTC AGC GTT ACT TCT TCG ATG C	**475**
	Rc.rompB.4,836n	CCG TAC TCC ATC TTA GCA TCA G	
	Rc.rompB.4,496p	CCA ATG GCA GGA CTT AGC TAC T	**267**
	Rc.rompB.4,762n	AGG CTG GCT GAT ACA CGG AGT AA	
*17kDa*	Rr17kDa.61p	GCT CTT GCA ACT TCT ATG TT	**434**
	Rr17kDa.492n	CAT TGT TCG TCA GGT TGG CpG	
*gltA*	RH314	AAA CAG GTT GCT CAT CAT TC	**857**
	CSF-R	AAG TAC CGT GAA CAT TTG CGA	
	CS-Ric-R	CAG TGA ACA TTT GCG ACG GTA	**852**
	CS535d	GCA ATG TCT TAT AAA TAT TC	Sequencing primer

FAM = fluorescein amidite.

### Statistical analysis

The Fischer’s exact test was used to compare the proportions between the *Rickettsia* seropositive and seronegative group concerning symptoms, as well as to examine the differences in seroreactivity between the geographical areas. The statistical analyses were conducted using IBM SPSS Statistics version 21. A p-value ≤ 0.05 (two-tailed) was considered statistically significant.

### Ethics statement

The study was reviewed and approved by the Regional Ethical Review Board, Linköping University (M132-06), and by the Ethics Committee of the Åland Health Care, 2008-05-23

## Results

Of the 218 participants included in the study, 82 (37.6%) were men and 136 (62.4%) women. The median age was 64 years (range 19 to 92), with 66 years for men (range 34 to 87) and 63 years for women (range 19 to 92). One hundred and four (47.7%) of the participants reported tick bites earlier in the season before enrolment, and 129 (59.2%) reported tick bites during the 3-month interval between inclusion and follow-up.

At follow-up, serological results regarding *Rickettsia* showed that 96 of 218 participants (44.0%) had IgG equal to or higher than the cut-off titre of 1:64. Out of the 96 participants, 40 (18.3%) seroconverted with a titre of at least 1:128 or showed a four-fold increase in IgG titre (Group 1), another four (1.8%) showed a single titre equal to or above 1:256 (Group 2), 52 (23.8%) had an IgG titre between 1:64 and 1:128 (Group 3), and 122 (56.0%) were seronegative (Group 4). In summary, 44 (20.2%) participants showed either a seroconversion with a four-fold increase in titre or a titre ≥ 1:256, thus indicating a recent or current infection (Tables 2–4).

**Table 2:** Distribution of symptoms for the combination rickettsia DNA positive tick and 4-fold rise in titre (seroconversion).

Area/P. no	Ric-DNA in tick/ tickstage	copies/μl reaction	Duration tick feeding (h)	SC	Ric/s-S1		Ric/s-S2	Self-reported symptoms	EM	Bo/s
					IgG	IgM	IgG			
K / (K56)	+ / Ny	8227	34	+	<1/64	1/64	1/256	h, f, fe, np, n v, am	pos	-
V / (L56)	+ / Ad	2	ND	+	<1/64	<1/64	1/128	h, am	-	-
Ö / (S69)	+ / Ny	210	ND	+	<1/64	<1/64	1/128	-	-	+
Ö / (S71)	+ / Ny	54674	24	+	<1/64	<1/64	1/128	-	-	+
Ö / (V6)	+ / Ny	852	52	+	<1/64	<1/64	1/256	-	-	-

P.no = participant number; SC = seroconversion; h = Headache; f = Fatigue; fe = Fever; np = neck pain; n = nausea; v = vertigo; am = arthralgia/myalgia; EM = erythema migrans; La = larvae; Ny = nymph; Ad = adult; Ric = rickettsia; h = hours; ND = no data; Ric/s = Rickettsia serology; dS1/S2 = Serum 1/Serum 2; Bo/s = Borrelia serology.

**Table 3:** Distribution of symptoms for the combination rickettsia DNA negative tick/s per participant and 4-fold rise in titre (seroconversion).

Area/P.no	Ric-DNA in tick/tickstage	Duration tick feeding (h)	Ric/s -S1		Ric/s -S2	Self-reported symptoms	EM	Bo/s
			IgG	IgM	IgG			
J / (B5)	- / ND	ND	<1/64	<1/64	1/128	-	-	+
J / (B47)	- / N	28	<1/64	<1/64	1/128	-	-	-
K / (K6)	- / N	<24	<1/64	<1/64	1/128	-	-	SC
K / (K7)	- / N	57	<1/64	<1/64	1/256	h, f, np, n, v, r, a, nu	-	-
K / (K8)	- / N	49	<1/64	<1/64	1/128	f, fe, np, v, c, r, a, nu	-	-
K / (K9)	- / N	24	1/64	1/64	1/256	-	-	+
K / (K11)	- / La	ND	<1/64	<1/64	1/128	v	-	+
K / (K12)	- / N	29	<1/64	<1/64	1/128	-	-	+
K / (K16)	- / N	24	<1/64	1/64	1/128	-	-	+
K / (K17)	- / N	50	<1/64	<1/64	1/128	h	-	-
K / (K20)	- / A	44	<1/64	1/256	1/128	f, fe, la, v, c, r, a, nu	+*	-
K / (K38)	- / N	29	<1/64	<1/64	1/128	-	-	-
K / (K43)	- / N	24	<1/64	<1/64	1/128	-	-	+
K / (K46)	- / A	34	1/64	1/256	1/256	f, fe, v	+*	-
K / (K48)	- / N	39	<1/64	<1/64	1/128	n, a	-	-
K / (K57)	- / N	24	<1/64	<1/64	1/128	-	-	-
V / (L13)	- / N	33	<1/64	<1/64	1/128	-	-	-
V / (L16)	- / N	27	<1/64	<1/64	1/256	-	-	SC
V / (L31)	- / N	47	<1/64	<1/64	1/128	-	-	+
V / (L38)	- / N	24	<1/64	</64	1/128	-	-	-
V / (L43)	- / N	24	1/64	<1/64	1/256	-	-	SC
V / (L46)	- / N	46	1/64	1/128	1/256	-	-	-
V / (L54)	- / N	24	<1/64	1/256	1/128	-	-	+
Ö / (S72)	- / A	ND	1/64	<1/64	1/256	h,np,w,a,nu	-	-
Ö / (S75)	- / A	34	1/64	<1/64	1/256	-	-	-
Ö / (V1)	- / N	24	1/64	1/128	1/256	-	-	+
Ö / (V19)	- / ND	ND	<1/64	1/64	1/128	-	-	-
Å / (Å2)	- / N	24	<1/64	1/256	1/128	-	-	+
Å / (Å3)	- / N	24	<1/64	1/128	1/128	-	-	-
Å / (Å11)	- / N	44	<1/64	1/64	1/128	-	-	-
Å / (Å16)	- / N	24	<1/64	1/256	1/256	-	-	+
Å / (Å19)	- / N	30	<1/64	<1/64	1/128	h, f, n, np, v, c	-	+
Å / (Å23)	- / N	46	1/64	1/128	1/256	-	-	-
Å / (Å31)	- / A	24	<1/64	<1/64	1/128	-	-	-
Å / (Å35)	- / N	24	<1/64	<1/64	1/256	-	-	+
Å / (Å36)	- / N	24	<1/64	<1/64	1/128	h, f, np, la, n, w, v, c, r, am, nu		

P.no = participant number; SC = seroconversion; h = Headache; f = Fatigue; fe = Fever; np = neck pain; la = loss of appetite; n = nausea; w = weight loss; v = vertigo; c = concentration difficulties; r = radiating pain; am = arthralgia / myalgia; nu = numbness; EM = erythema migrans; La = larvae; Ny = nymph; Ad = adult; Ric = rickettsia; h = hours; ND = no data; Ric/s = Rickettsia serology; S1/S2 = Serum 1/Serum 2;Bo/s = Borrelia serology;

* Not related to tick-borne-disease.

**Table 4:** Distribution of symptoms for the combination rickettsia DNA negative tick/s per participant and IgG titre ≥ 1:256.

Area/P.no	Ric- DNA in tick/stage	Duration tickfeeding (h)	IgG titre ≥1:256	Ric/s S1		Ric/s S2	Self-reported symptoms	EM	Bo/s
				IgG	IgM	IgG			
K / (K28)	- / Ny	26	+	1/256	ND	1/128	np, r, am, nu	-	+
K / (K55)	- / Ny	ND	+	1/256	ND	1/128	-	-	-
VG / (L4)	- / Ny	42	+	1/128	ND	1/256	-	-	+
Å / (Å13)	- / Ny	41	+	1/128	ND	1/256	-	-	+

P.no = participant number; Np = neck pain; R = radiating pain; Am = arthralgia / myalgia; Nu = numbness; EM = erythema migrans; L< = larvae; Ny = nymph; Ad = adult; Ric = rickettsia; h = hours; ND = no data; Ric/s = Rickettsia serology; S1/S2 = Serum 1/Serum 2; Bo/s = Borrelia serology: VG = Västra Götaland; Ö = Östergötland; J = Jönköping County; K = Kalmar County.

**Fig 2:**
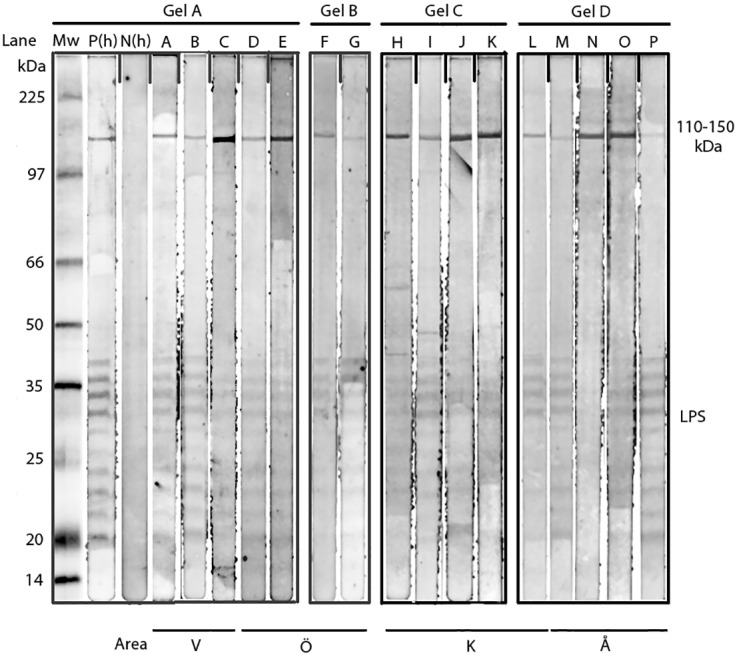
Western Blot analysis of IgG antibodies against *R*. *helvetica* whole cell antigen. Lane A-P demonstrates the lipopolysaccaride ladders and specific reactions against *R*. *helvetica* proteins in the 110-150-kDa region for serum 2 for patients (Lane) V16(A), V43(B), V46 (C)(Area V); S71(D), S72(E), S75(F), V6(G) (Area Ö); K7(H), K9(I), K14(J), K46(K) K56(L) (Area K); Å13(M), Å16(N), Å23(O), Å35(P) (Area Å) in titres 1:200. Lane P(h) demonstrates specific proteins and the lipopolysaccharide (LPS) ladders reacting with a human antiserum from a patient diagnosed with rickettsial infection and N(h) a healthy negative blood donor. Mw = molecular weight marker. “Fig 2” is compiled of four figure panels representing the groups of lanes that originated from different gels/blots (Gel A-D). The short vertical lines of “Fig 2” divide the individual non-adjacent lanes in the gels. The original analyses are presented in S1–S4 Figs with Gels A-D as Supporting Information.

WB for patient’s nos. K7, K9, K46, K56, L16, L43, L46, S71. S72, S75, V6, Å16, Å23 and Å35 (Group 1) and K13 and Å13 (Group 2) showed a specific response against lipopolysaccharide (LPS) and protein antigens in the 110–150 kDa region for IgG to whole cell antigen of *R*. *helvetica* (Fig 2). Negative controls in the form of serum from a healthy blood donor and IFA negative participant showed no specific reactions.

In the four regions representing south central and southernmost Sweden, there were no statistically significant differences between the groups (1–4) compared to the Åland Islands regarding serologic findings showing antibodies to *Rickettsia* spp. (Table 5). Of the collected 472 ticks that had bitten humans, 39 (8.3%) were positive for *Rickettsia*, as indicated by real-time PCR. All of these amplicons were further analysed using PCR targeting at least one of the genes encoding for *ompB*, citrate synthase (*gltA*) or the 17kDa protein followed by sequencing. Twenty-three amplicons showed a sequence with a 100% match to the deposited sequences in Gen Bank representing *R*. *helvetica*. The other 16 samples had weak signals in real-time PCR and showed no product by amplification with the other PCR assays. The ticks with confirmed infestation with *R*. *helvetica* were 11/98 (11.2%) from Lidköping, which was significantly higher than for the other areas, 4/98 (4.1%) from Östergötland, 7/97 (7.2%) from Jönköping, 1/82 (1,2%) from Kalmar and 0/98 (0%) from the Åland Islands. Of the ticks that were identified by stage, 90 were adults, 288 nymphs and 14 larvae and correlated with Group 1–4, as shown in Table 6.

**Table 5:** Serological outcomes (Groups 1–4) in relation to the geographical areas.

Area	Sero-Group 1 no/(%)	Sero-Group 2 no/(%)	Sero-Group 3 no/(%)	Sero-Group 4 no/(%)	Total = 218 no/(%)
VG+Ö+J+K	31 (17,3)	3 (1.7)	42 (23.5)	103 (57.5)	179 (100)
Åland Islands	9 (23.1)	1 (2.6)	10 (25.6)	19 (48.7)	39 (100)

no = number; VG = Västra Götaland; Ö = Östergötland; J = Jönköping County; K = Kalmar County

**Table 6:** Life cycle stages of ticks examined in relation to serologic Groups 1–4.

Life cycle stage	Sero-Group 1	Sero-Group 2	Sero-Group 3	Sero-Group 4	Total
Female adult	21	0	21	48	90
Nymph	91	4	117	76	288
Larvae	7	0	6	1	14
**Total**	**119**	**4**	**144**	**125**	**392**

Blood feeding duration was calculated in 327 ticks, 90 of which belonged to Group 1, four to Group 2, 117 to Group 3, and 116 to Group 4. The median blood feeding time was 27 hours (h) in Group 1, 32 h in Group 2, 32 h in Group 3 and 34 h in Group 4. Sixteen (7.3%) of the participants had been bitten by at least one *Rickettsia*-infected tick. Five of the 40 (12.5%) participants in Group 1 had been bitten by a *Rickettsia*-infected tick, no one in Group 2, seven of 52 (13.5%) in Group 3, and 3 of 122 (2.5%) in Group 4. Of the total of 16 participants bitten by a *Rickettsia*-infected tick, five (31.3%) seroconverted or had a four-fold increase in antibody titres. Of the 10 ticks in which *R*. *helvetica* was quantified by PCR, the mean value for Group 1 (4 ticks) was 12793 copies/μl reaction and for Group 4 (13 ticks) 35847 copies/μl. Symptoms of the participants bitten by rickettsial-positive ticks are listed in Table 7.

**Table 7:** Distribution of symptoms for the combination rickettsia DNA positive tick/s per participant and seroreactivity, but no 4-fold rise in titre (seroconversion).

Area/P.no	Ric-DNA in tick/tick stage	Ric copies/ μl reaction	Duration tick-feeding (h)	Ric/s-S1		Ric/s-S2	Self-reported symptoms	EM	Bo/s
				IgG	IgM	IgG			
J / (B6)	+ / A	36697	<24	ND	ND	<1/64	-	-	-
J / (B21)	+ / N	33454	<24	ND	ND	1/64	-	-	-
K / (K50)	+ /A & N	890/12	ND & 35	ND	ND	1/64	fa, r, am, nu	-	+
K / (K136)	+ / N	23344	ND	ND	ND	1/64	fa, np, r, am, nu	-	-
L / (L2)	+ /N & N	9420/87	26 & 53	ND	ND	1/64	-	-	-
L / (L17)	+ / N		26	ND	ND	<1/64	-	-	-
L / (L28)	+ /N & A	95000/ 110000	33 & 35	ND	ND	1/64	-	-	-
L / (L33)	+ / A	8	<24	ND	ND	1/64	h, fa, np, n, v, am	-	+
L / (L40)	+ / N	10631	81	ND	ND	<1/64	-	-	-
Ö / (V15)	+ / A	902	53	ND	ND	<1/64	h, fa, r, nu	-	+
Ö / (V27)	+ / A	145566	<24	1/64	ND	1/128	-	-	-

P.no = participant no; SC = seroconversion; h = Headache; fa = Fatigue; np = neck pain; n = nausea; v = vertigo; r = radiating pain; am = arthralgia / myalgia; nu = numbness; EM = erythema migrans; L = larvae; N = nymph; A = adult; Ric = rickettsia; h = hours; ND = no data; Ric/s = Rickettsia serology; S1/S2 = Serum 1/Serum 2; Bo/s = Borrelia serology; VG = Västra Götaland; Ö = Östergötland; J = Jönköping County; K = Kalmar County

Forty-seven (21.6%) of the participants self-reported at least one non-specific symptom at the follow-up visit (Table 8). The symptoms were consistently unspecific, and only a few participants sought medical care for their symptoms, though none was diagnosed with tick-borne diseases. Most of the symptoms were equally distributed in all groups, but nausea (p = 0.006) and radiating pain (p = 0.041) were more common in Group 1 and 2 combined than in Group 4. Only three participants developed erythema migrans (EM), one each in Group 1, 3 and 4 (Table 8). Only the participant in Group 1 had been bitten by a rickettsial-positive tick. In this case, *Borrelia* serology was negative. In Group 3, the participant was seroreactive for both *Borrelia* and *Rickettsia*, but did not seroconvert. The participant in Group 4 seroconverted to *Borrelia*. Among those who seroconverted (Group 1) and were bitten by a *Rickettsia* spp.-positive tick, 20.0% reported more than three non-specific symptoms (Table 2). The corresponding percentage for participants who seroconverted and had been bitten by a *Rickettsia* spp.-negative tick was 16.7%, compared to 42.9% for participants who showed seroreactivity and also had been bitten by a *Rickettsia* spp.-positive tick (Tables 3 and 7). For those who were seronegative (Group 4), only 10.7% reported more than three non-specific symptoms. The participants who showed seroreactivity and had been bitten by a *Rickettsia* spp.-positive tick (Table 7) had significantly more non-specific symptoms compared to the seronegative group (Group 4) (p = 0.041).

**Table 8:** Self-reported non-specific symptoms and results of *Rickettsia* spp. serology in relation to outcome in Serogroups 1–4.

Symptoms	Sero-Group 1 No/(%)	Sero-Group 2 No/(%)	Sero- Group 3 No/(%)	Sero-Group 4 No/(%)	Total No/(%)
Headache	6 (15.0)	0 (0.0)	7 (13.5)	17 (13.9)	30 (13.8)
Fatigue	7 (17.5)	0 (0.0)	9 (17.3)	14 (11.5)	30 (13.8)
Fever	4 (10.0)	0 (0.0)	1 (1.9)	2 (1.6)	7 (3.2)
Neck pain	4 (10.0)	1 (25.0)	5 (9.6)	10 (8.2)	20 (9.2)
Loss of appetite	3 (7.5)	0 (0.0)	0 (0.0)	5 (4.1)	8 (3.7)
Nausea	5 (12.5)	0 (0.0)	3 (5.8)	1 (0.8)	9 (4.1)
Weight loss	1 (2.5)	0 (0.0)	0 (0.0)	2 (1.6)	3 (1.4)
Vertigo	7 (17.5)	0 (0.0)	2 (3.8)	10 (8.2)	19 (8.7)
Cognitive difficulties	4 (10.0)	0 (0.0)	2 (3.8)	5 (4.1)	11 (5.0)
Radiating pain	5 (12.5)	1 (25.0)	4 (7.7)	4 (3.3)	14 (6.4)
Myalgia/arthralgia	8 (20.0)	1 (25.0)	9 (17.3)	11 (9.0)	29 (13.3)
Numbness	5 (12.5)	1 (25.0)	4 (7.7)	7 (5.7)	17 (7.8)
Erythema migrans	1 (2.5)	0 (0.0)	1 (1.9)	1 (10.8)	3 (1.4)
**Total**	**40 (18.3)**	**4 (1.8)**	**52 (23.9)**	**122 (56.0)**	**218 (100)**
*Borrelia* Seroconversion	3 (7.5)	0 (0.0%)	0 (0.0)	4 (3.3)	7 (3.2)
*Borrelia* seropositive	17 (42.5)	3 (75.0%)	24 (46.2)	44 (36.1)	88 (40.4)

Ninety-five (43.6%) of the participants were seropositive to *Borrelia* spp. Of these seven participants (3.2%) showed seroconversion against *Borrelia* spp. (Table 8). Three of them belonged to Group 1; 1.4% seroconverted to both *Rickettsia* spp. and *Borrelia* spp., and four belonged to Group 4. In addition, 88 participants (40.4%) were seropositive to *Borrelia* spp., of whom 17 of 40 (42.5%) were positive in Group 1, three of 4 (75.0%) in Group 2, 24 of 52 (46.2%) in Group 3, and 44 of 122 (36.1%) in Group 4.

## Discussion

The current study presents the prevalence of *Rickettsia* sp. in ticks, serological results and symptoms on tick-bitten individuals in Sweden and on the Åland Islands. All amplicons that were sequenced represented *R*. *helvetica* sequences. Previous studies in Sweden have shown a prevalence of *Rickettsia*-positive ticks collected from nature of between 1.7% and 22.1%, with a median between 9% and 11% [2, 3]. The present study supports previous findings showing that *R*. *helvetica* is the most prevalent *Rickettsia* sp. and, besides an occasional finding of *R*. *slovaca*, thus far the only reported tick-borne *Rickettsia* sp. to have infected humans in Sweden. Seroconversion after tick-bites should therefore primarily reflect antibodies to *R*. *helvetica*.

The results are in accordance with findings from previous surveys in high endemic areas in Sweden. In a previous study of recruits in Sweden, a four-fold increase in titre or seroconversion was found in 22.9% of participants [6]. Although there were fewer participants in that study, the results are similar to the present findings and probably represent a baseline for what can be expected in individuals frequently exposed to ticks in Sweden.

Seroconversion to *Rickettsia* spp. was more common than seroconversion to *Borrelia* spp., suggesting that, in these areas, the risk of becoming infected with *Rickettsia* spp. after a tick bite is higher than for *Borrelia* spp. Ninety-five (43.6%) of the participants were either seropositive or showed seroconversion to *Borrelia* spp., compared to 96 (44.0%) for *Rickettsia* spp. Seroconversion was more common to *Rickettsia* spp. than to *Borrelia* spp., but the number of participants with antibodies to both agents was equal. Antibodies to *Borrelia* can remain for years while antibodies to *Rickettsia* seem to remain for a shorter period, where IgG can persist for at least 8–12 months [31–33]. The latter may be of importance in explaining why participants with a recent or current co-infection with *Borrelia* spp. and *Rickettsia* spp., based on seroconversion, were fewer in the present study compared to findings from earlier studies where co-infection was based on sero-reactivity [18, 20].

The blood feeding time required for transmission of rickettsial bacteria to the vertebrate host has been reported to be between 10 minutes and ≥10 hours [34]. The blood feeding time in our study was almost equal in all groups (1–4), with a median time over 24 hours. A longer blood feeding time did not correlate with a higher infection rate, which is consistent with previously reported data [34]. When measuring the number of bacteria infesting the ticks, using PCR quantification, lower numbers of rickettsia DNA copies per μl reaction were found in Group 1 than in Group 4. According to these results, there was no correlation between higher numbers of DNA copies/μl and seroconversion.

Surprisingly, none of the ticks collected from the Åland Islands contained *Rickettsia* spp., although the participants showed the same percentage of seroconversion and seroreactivity as those in the other study areas. All participants from the Åland Islands reported having acquired their tick bites in that area. The Åland Islands are known to be a very high endemic area for ticks, and the participants from this area had an average of 2.7 ticks collected per person in comparison to 1.7 ticks collected per person among the Swedish participants. The explanation could be that although the prevalence of *Rickettsia* spp. in ticks on the Åland Islands is lower than in the Swedish areas, the citizens of Åland get more tick bites and thus an equal amount of exposure to *Rickettsia* spp. ticks as the other study participants.

Most of the ticks in Group 1 were not infected by *Rickettsia* spp, but the absence of ticks positive does not imply that the person was not bitten by another overlooked positive tick. For this reason, it seems likely that these participants had been bitten by a number of ticks during the season and that the ticks collected at study enrolment were not the ticks that caused the infection. Therefore, based on the present data, it is difficult to draw conclusions concerning the exact risk of becoming infected when bitten by a *Rickettsia*-positive tick.

Regarding self-reported non-specific symptoms, we only found that nausea and radiating pain were more common among participants in Group 1 and 2 combined compared to those in Group 4. Among the participants with several non-specific symptoms, those who showed seroreactivity and had been bitten by a *Rickettsia* spp.-positive tick had a greater number of non-specific symptoms than did the seronegative participants (Group 4). However, the groups were small, and the self-reporting of symoptims may not always be accurate, meaning that no certain conclusions can be drawn. EM was not common and the participants presenting with EM were few, why any conclusion regarding the possibility that EM could be caused by tick-borne agents other than *Borrelia* spp. cannot be drawn.

### Conclusion

Our findings on clinical manifestations support the assumption that *R*. *helvetica* is primarily a subclinical disease with non-specific symptoms, likely leading to an under-estimation of human cases. However, severe symptoms may develop as well (meningitis, septicaemia and myocarditis) and be misinterpreted as another tick-borne disease. More knowledge about the course and frequency of the infection is needed but the present study shows that rickettsial infection is a common tick-borne infection in Sweden and should be considered among tick-bitten persons.

## Supporting Information

S1 Fig Gel A. Patients(lane) V16(A), V43(B), V46 (C); S71(D), S72(E). Western Blot analysis of IgG antibodies against *R*. *helvetica* whole cell antigen for serum 2 in titres 1:200. Mw = molecular weight marker. P(h) and N(h) represent positive and negative human control sera.
(TIF)

S2 Fig Gel B. Patients(lane) S75(F), V6(G). x-s = extra serum. Western Blot analysis of IgG antibodies against *R*. *helvetica* whole cell antigen for serum 2 in titres 1:200. Mw = molecular weight marker. P(h) and N(h) represent positive and negative human control sera.
(TIF)

S3 Fig Gel C. Patients(lane) K7(H), K9(I), K14(J), K46(K), K55(Table 4). Western Blot analysis of IgG antibodies against *R*. *helvetica* whole cell antigen for serum 2 in titres 1:200. Mw = molecular weight marker. P(h) and N(h) represent positive and negative human control sera.
(TIF)

S4 Fig Gel D. Patients(lane) K56(L); Å13(M), Å16(N), Å23(O), Å35(P). Western Blot analysis of IgG antibodies against *R*. *helvetica* whole cell antigen for serum 2 in titres 1:200. Mw = molecular weight marker. P(h) and N(h) represent positive and negative human control sera.
(TIF)

## References

[1] Parola P, Paddock CD, Socolovschi C, Labruna MB, Mediannikov O, Kernif T, et al Update on tick-borne rickettsioses around the world: a geographic approach. Clin Microbiol Rev. 2013; 26: 657–702. 10.1128/CMR.00032-13 24092850 PMC3811236

[2] Wallmenius K, Pettersson JH, Jaenson TG, Nilsson K. Prevalence of *Rickettsia* spp., *Anaplasma phagocytophilum*, and *Coxiella burnetii* in adult *Ixodes ricinus* ticks from 29 study areas in central and southern Sweden. Ticks Tick Borne Dis. 2012; 3:100–106. 10.1016/j.ttbdis.2011.11.003 22487426

[3] Severinsson K, Jaenson TG, Pettersson J, Falk K, Nilsson K. Detection and prevalence of *Anaplasma phagocytophilum* and *Rickettsia helvetica* in *Ixodes ricinus* ticks in seven study areas in Sweden. Parasit Vectors. 2010; 3: 66, 10.1186/1756-3305-3-66 20684755 PMC2923137

[4] Fournier P, Allombert C, Supputamongkol Y, Caruso G, Brouqui P, Raoult D. Aneruptive fever associated with antibodies to *Rickettsia helvetica* in Europe and Thailand. J of Clin Microbiol. 2004; 42:816–818. 14766859 10.1128/JCM.42.2.816-818.2004 PMC344501

[5] Phongmany S, Rolain J, Phetsouvanh R, Blacksell SD, Soukkhaseum V, Rasachack B, et al Rickettsial infections and fever, Vientiane, Laos. Emerg Inf Dis. 2006; 12: 256–262. 10.3201/eid1202.050900 PMC3373100 16494751

[6] Nilsson K, Lukinius A, Påhlson C, Moron C, Hajem N, Olsson B, et al Evidence of *Rickettsia* spp. infection in Sweden: a clinical, ultrastructural and serological study. APMIS. 2005; 113:126–134. 10.1111/j.1600-0463.2005.apm1130206.x 15723687

[7] Nilsson K. Septicaemia with *Rickettsia helvetica* in a patient with acute febrile illness, rash and myasthenia. J of Inf. 2009; 58: 79–82. 10.1016/j.jinf.2008.06.005 18649945

[8] Nilsson K, Lindquist O, Pahlson C (1999) Association of *Rickettsia helvetica* with chronic perimyocarditis in sudden cardiac death. Lancet. 1999: 354:1169–1173. 10.1016/S0140-6736(99)04093-3 10513711

[9] Nilsson K, Elfving K, Pahlson C.*Rickettsia helvetica* in patient with meningitis, Sweden, 2006. Emerg Inf Dis.2010; 16:490–492. 10.3201/eid1603.090184 PMC3322002 20202426

[10] Nilsson K, Wallménius K, Pahlson C. Coinfection with *Rickettsia helvetica* and Herpes Simplex Virus 2 in a Young Woman with Meningoencephalitis. Case Rep Infect Dis. 2011:469194 10.1155/2011/469194. Epub 2011 Oct 19. 22567472 PMC3336230

[11] Nilsson K, Wallménius K, Hartwig S, Norlander T, Pahlson C. Bell's palsy and sudden deafness associated with *Rickettsia* spp. infection in Sweden. A retrospective and prospective serological survey including PCR findings. Eur J Neurol. 2014;,21:206–214. 10.1111/ene.12218 23790098 PMC4232316

[12] Portillo A, Santibanez S, Garcia-Alvarez L, Palomar AM, Oteo JA. (2015) Rickettsioses in Europe. Microbes Infect. 2015; 17: 834–838. 10.1016/j.micinf.2015.09.009 26384814

[13] Lindblom A, Severinson K, Nilsson K. *Rickettsia felis* infection in Sweden: report of two cases with subacute meningitis and review of the literature. Scand J Inf Dis. 2010; 42: 906–909. 20735330 10.3109/00365548.2010.508466

[14] Wallménius K, Barboutis C, Fransson T, Jaenson TG, Lindgren PE, Nystrom F, et al Spotted fever *Rickettsia* species in *Hyalomma* and *Ixodes* ticks infesting migratory birds in the European Mediterranean area. Parasit Vectors 2014; 7: 318 10.1186/1756-3305-7-318 25011617 PMC4230250

[15] Milhano N, de Carvalho IL, Alves AS, Arroube S, Soares J, Rodriguez P, et al Coinfections of *Rickettsia slovaca* and *Rickettsia helvetica* with *Borrelia lusitaniae* in ticks collected in a Safari Park, Portugal. Ticks Tick Borne Dis 2010; 1:172–177. 10.1016/j.ttbdis.2010.09.003 21771525

[16] May K, Strube C. Prevalence of Rickettsiales (*Anaplasma phagocytophilum* and *Rickettsia* spp.) in hard ticks (*Ixodes ricinus*) in the city of Hamburg, Germany. Parasitology Research 2014;113: 2169–2175. 10.1007/s00436-014-3869-x 24728556

[17] Mancini F, Di Luca M, Toma L, Vescio F, Bianchi R, Khoury C, et al Prevalence of tick-borne pathogens in an urban park in Rome, Italy. Ann Agric Environ Med. 2014; 21: 723–727. 10.5604/12321966.1129922 25528909

[18] Lindblom A, Wallménius K, Nordberg M, Forsberg P, Eliasson I, Pahlson C, et al Seroreactivity for spotted fever rickettsiae and co-infections with other tick-borne agents among habitants in central and southern Sweden. Eur J Clin Microbiol Infect Dis. 2013; 32: 317–323. 10.1007/s10096-012-1742-3 22961007 PMC3569577

[19] Tijsse-Klasen E, Sprong H, Pandak N (2013) Co-infection of *Borrelia burgdorferi* sensu lato and *Rickettsia* species in ticks and in an erythema migrans patient. Parasit Vectors. 2013; 6:347 10.1186/1756-3305-6-347 24326096 PMC3878868

[20] Nogueras MM, Roson B, Lario S, Sanfeliu I, Pons I, Anton E, et al Coinfection with "*Rickettsia sibirica subsp*. *mongolotimonae*" and *Rickettsia conorii* in a Human Patient: a Challenge for Molecular Diagnosis Tools. J Clin Microbiol. 2015; 53: 3057–3062. 10.1128/JCM.00457-15 26135877 PMC4540899

[21] Swanson SJ, Neitzel D, Reed KD, Belongia EA. Coinfections acquired from ixodes ticks. Clin Microbiol Rev. 2006; 19:708–727. 10.1128/CMR.00011-06 17041141 PMC1592693

[22] Dubourg G, Socolovschi C, Del Giudice P, Fournier PE, Raoult D. Scalp eschar and neck lymphadenopathy after tick bite: an emerging syndrome with multiple causes. Eur J Clin Microbiol Infect Dis. 2014; 33: 1449–1456. 10.1007/s10096-014-2090-2 24682865

[23] Koetsveld J, Tijsse-Klasen E, Herremans T, Hovius JW, Sprong H. Serological and molecular evidence for spotted fever group *Rickettsia* and *Borrelia burgdorferi* sensu lato co-infections in The Netherlands. Ticks Tick Borne Dis. 2015; 7: 371–377. 10.1016/j.ttbdis.2015.12.010 26739030

[24] Wilhelmsson P, Fryland L, Lindblom P, Sjöwall J, Ahlm C, Berglund J, et al A prospective study on the incidence of *Borrelia burgdorferi* sensu lato infection after a tick bite in Sweden and on the Åland Islands, Finland (2008–2009). Ticks Tick Borne Dis. 2015; 7: 71–79. 10.1016/j.ttbdis.2015.08.009 26341726

[25] Fryland L, Wilhelmsson P, Lindgren PE, Nyman D, Ekerfelt C, Forsberg P. Low risk of developing *Borrelia burgdorferi* infection in the south-east of Sweden after being bitten by a *Borrelia burgdorferi*-infected tick. Int J Infect Dis. 2011; 15:e174–181. 10.1016/j.ijid.2010.10.006 21168354

[26] Wilhelmsson P, Lindblom P, Fryland L, Nyman D, Jaenson TG, Forsberg P, et al *Ixodes ricinus* ticks removed from humans in Northern Europe: seasonal pattern of infestation, attachment sites and duration of feeding. Parasit Vectors. 2013;6:362 10.1186/1756-3305-6-362 24360096 PMC3880168

[27] Wilhelmsson P, Lindblom P, Fryland L, Ernerudh J, Forsberg P, Lindgren PE. Prevalence, diversity, and load of Borrelia species in ticks that have fed on humans in regions of Sweden and Aland Islands, Finland with different Lyme borreliosis incidences. PloS One. 2013;8(11):e81433 10.1371/journal.pone.0081433 24278437 PMC3836827

[28] Gray J, Stanek G, Kundi M, Kocianova E. Dimensions of engorging *Ixodes ricinus* as a measure of feeding duration. Int J Med Microbiol. 2005; 295: 567–572. 10.1016/j.ijmm.2005.05.008 16325552

[29] Brouqui P, Bacellar F, Baranton G, Birtles RJ, Bjoersdorff A, Blanco JR, et al Guidelines for the diagnosis of tick-borne bacterial diseases in Europe. Clin Microbiol & Inf. 2004; 10: 1108–1132. 10.1111/j.1469-0691.2004.01019.x 15606643

[30] Raoult D. and Dasch G.A., Immunoblot cross-reactions among *Rickettsia*, *Proteus* spp. and *Legionella* spp. in patients with Mediterranean spotted fever. FEMS Immunol Med Microbiol, 1995 11(1): p. 13–8. 7541270 10.1111/j.1574-695X.1995.tb00073.x

[31] Hammers-Berggren S, Lebech A-M, Karlsson M, Svenungson B, Hansen K, Stiernstedt G. Serological Follow-up after treatment of patients with erythema migrans and neuroborreliosis. J Clin Microbiol. 1994; 32: 1519–1525. 8077398 10.1128/jcm.32.6.1519-1525.1994 PMC264030

[32] Kalish RA, McHugh G, Granquist J, Shea B, Ruthazer R, Steere AC. Persistence of immunoglobulin M or immunoglobulin G antibody responses to *Borrelia burgdorferi* 10–20 years after active Lyme disease. Clin Infect Dis. 2001; 33:780–785. 10.1086/322669 11512082

[33] Clements ML, Dumler JS, Fiset P, Wisseman CL Jr., Snyder MJ, Levine MM, et al Serodiagnosis of Rocky Mountain spotted fever: comparison of IgM and IgG enzyme-linked immunosorbent assays and indirect fluorescent antibody test. J Inf Dis. 1983; 148: 876–880. 6415180 10.1093/infdis/148.5.876

[34] Saraiva DG, Soares HS, Soares JF, Labruna MB. Feeding period required by *Amblyomma aureolatum* ticks for transmission of *Rickettsia rickettsii* to vertebrate hosts. Emerg Inf Dis. 2014; 20:1504–1510. 10.3201/eid2009.140189 PMC4178383 25148391

