# Supplementary figures and images for: Prevalence of Rickettsia spp. in Ticks and Serological and Clinical Outcomes in Tick-Bitten Individuals in Sweden and on the Åland Islands
Source: PLoS One. 2016 Nov 15;11(11):e0166653. doi: 10.1371/journal.pone.0166653 (PMC5113005; doi:10.1371/journal.pone.0166653)

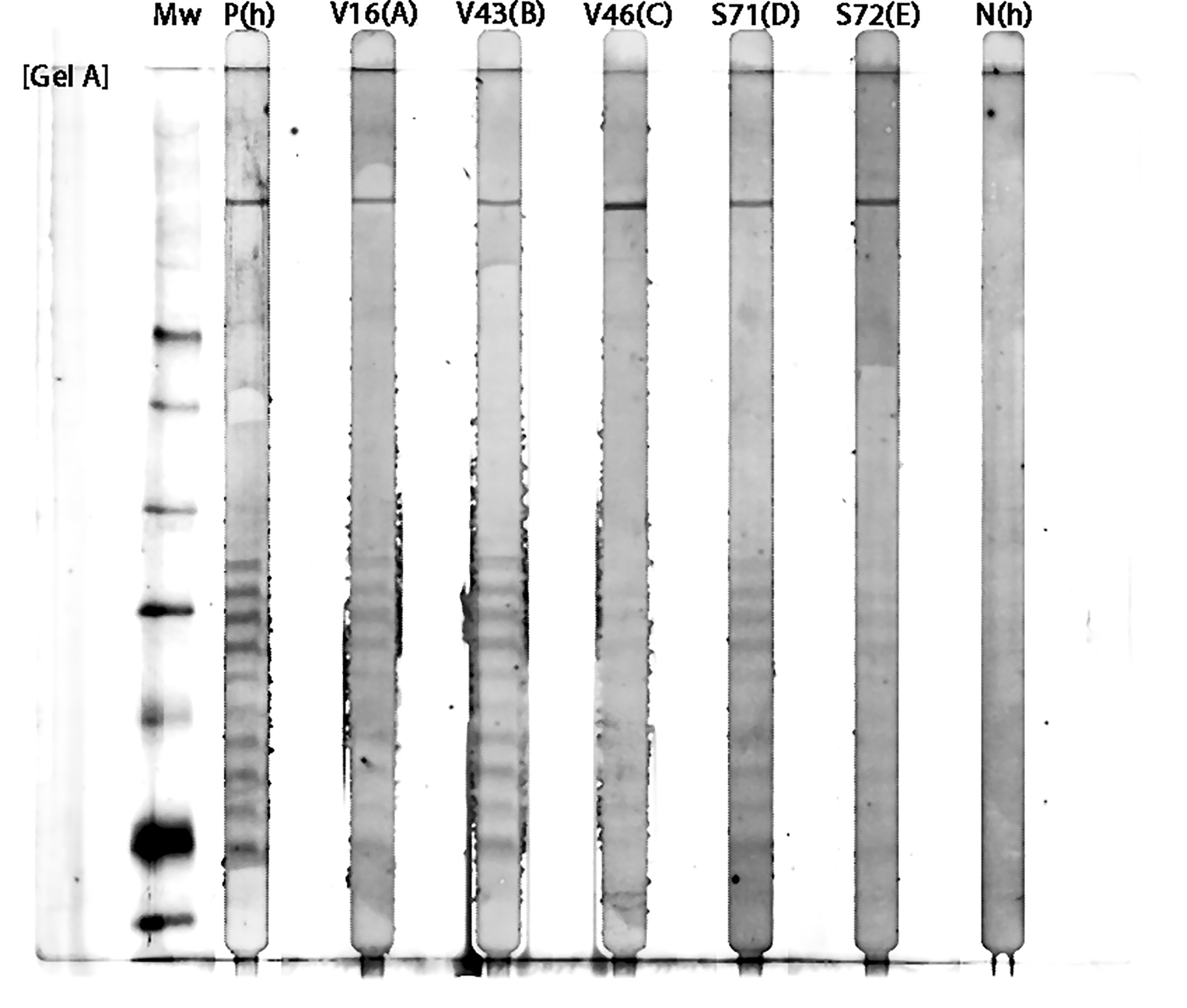

Supplement: S1 Fig — Patients(lane) V16(A), V43(B), V46 (C); S71(D), S72(E). Western Blot analysis of IgG antibodies against R. helvetica whole cell antigen for serum 2 in titres 1:200. Mw = molecular weight marker. P(h) and N(h) represent positive and negative human control sera. (TIF) [file pone.0166653.s001.tif]

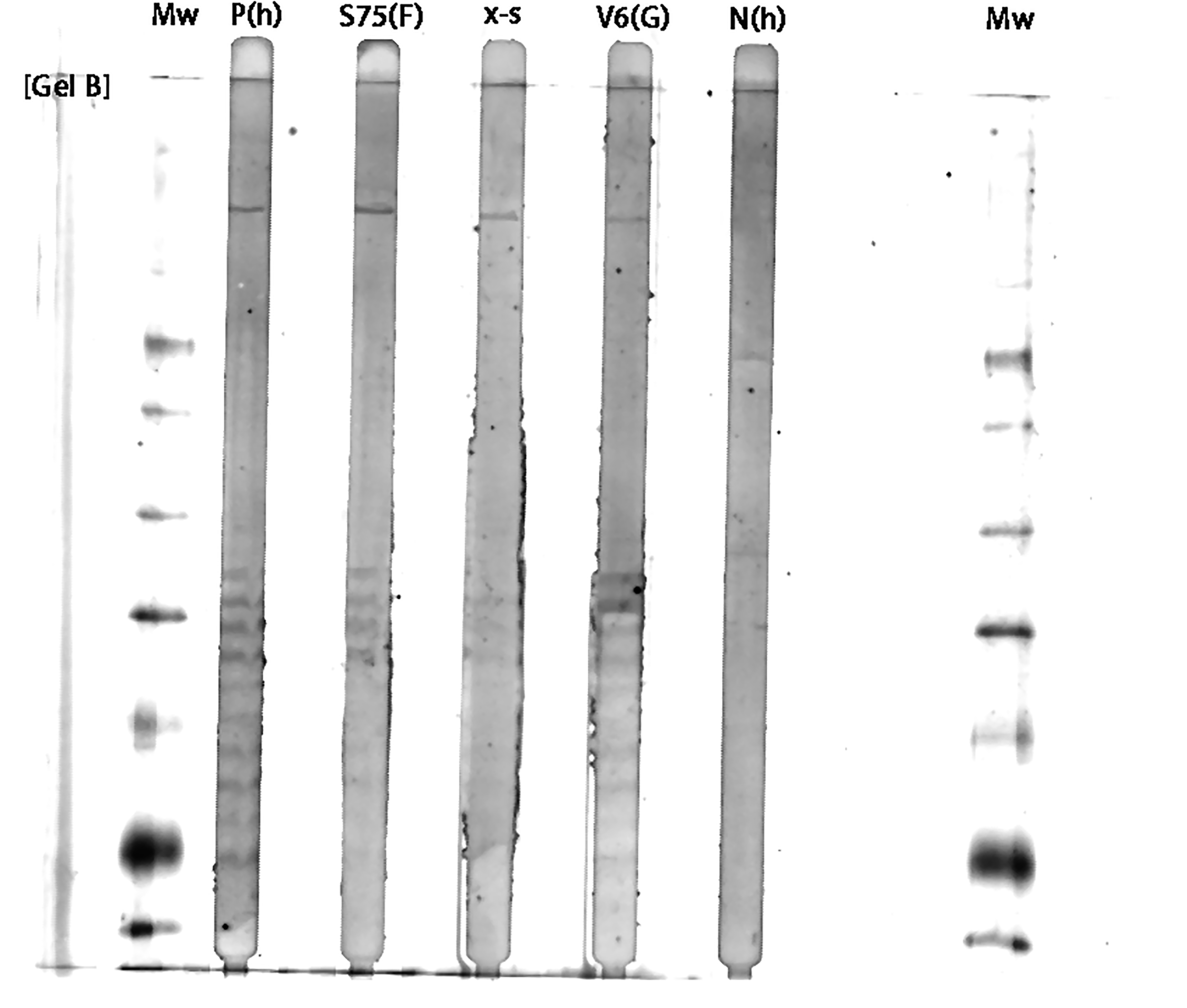

Supplement: S2 Fig — Patients(lane) S75(F), V6(G). x-s = extra serum. Western Blot analysis of IgG antibodies against R. helvetica whole cell antigen for serum 2 in titres 1:200. Mw = molecular weight marker. P(h) and N(h) represent positive and negative human control sera. (TIF) [file pone.0166653.s002.tif]

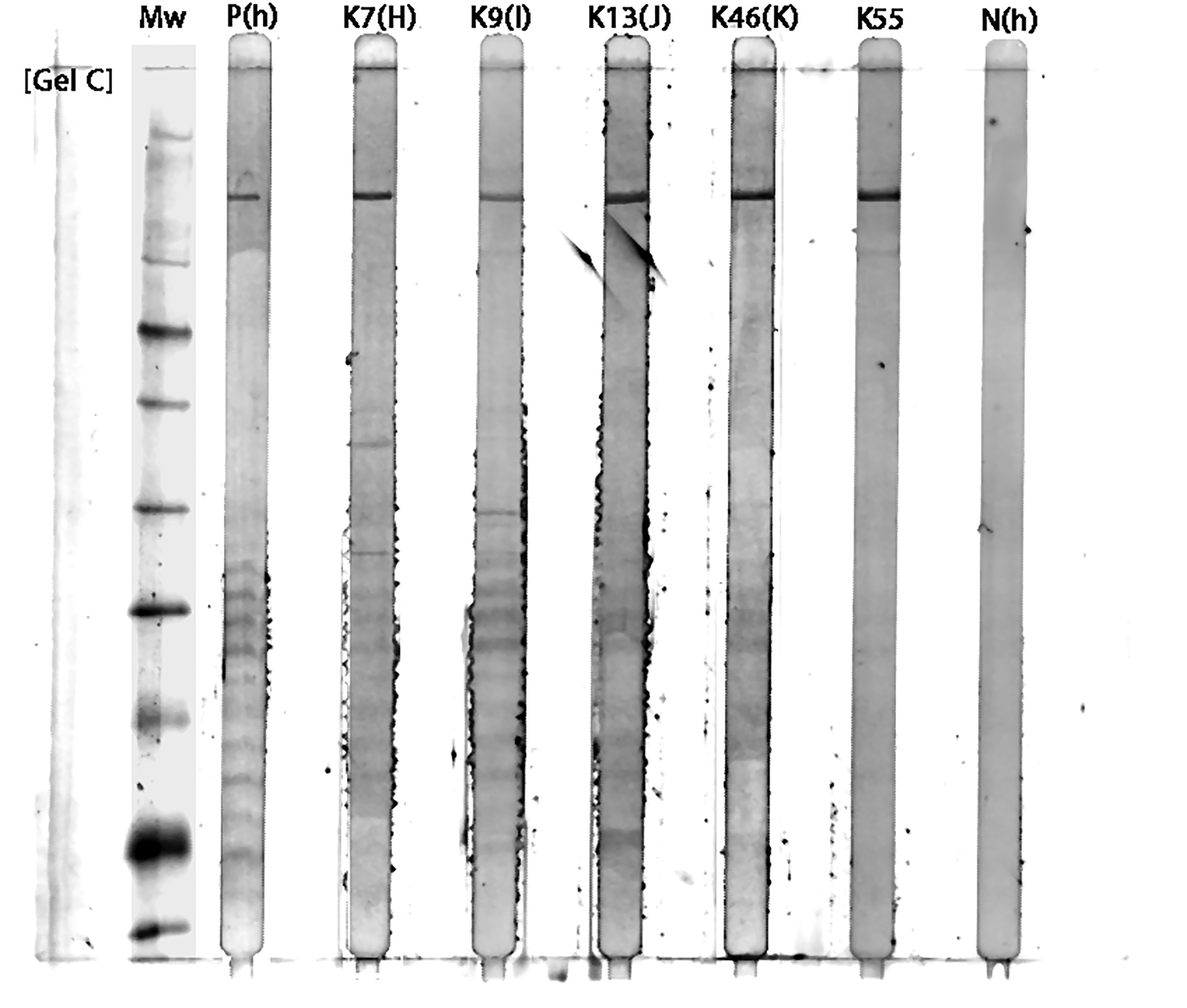

Supplement: S3 Fig — Patients(lane) K7(H), K9(I), K14(J), K46(K), K55(Table 4). Western Blot analysis of IgG antibodies against R. helvetica whole cell antigen for serum 2 in titres 1:200. Mw = molecular weight marker. P(h) and N(h) represent positive and negative human control sera. (TIF) [file pone.0166653.s003.tif]

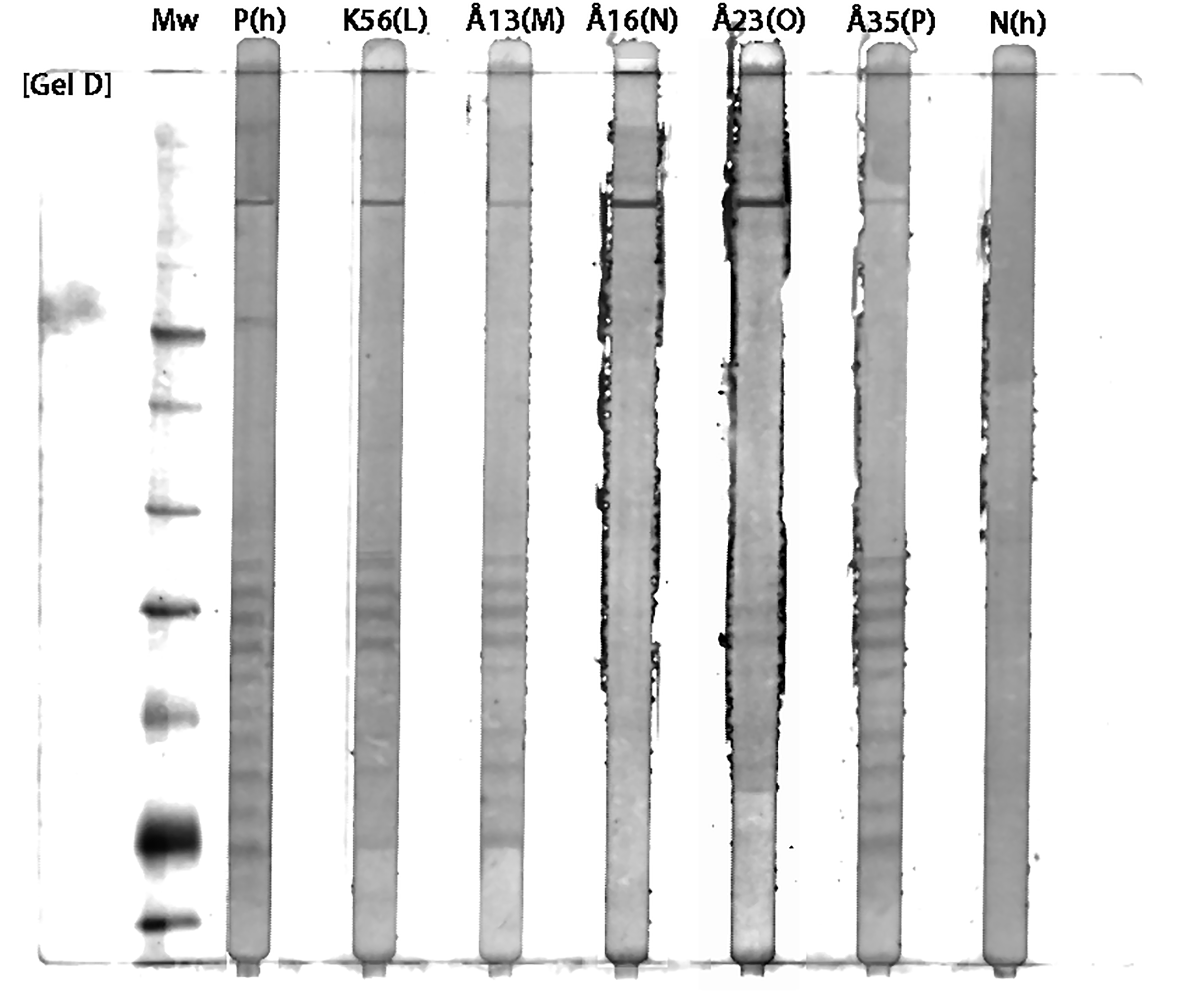

Supplement: S4 Fig — Patients(lane) K56(L); Å13(M), Å16(N), Å23(O), Å35(P). Western Blot analysis of IgG antibodies against R. helvetica whole cell antigen for serum 2 in titres 1:200. Mw = molecular weight marker. P(h) and N(h) represent positive and negative human control sera. (TIF) [file pone.0166653.s004.tif]
